# Mismatch repair gene mutations and cancer risks: an update

**DOI:** 10.1186/1897-4287-10-S2-A22

**Published:** 2012-04-12

**Authors:** AK Win, JL Hopper, MA Jenkins

**Affiliations:** 1 Centre for Molecular, Environmental, Genetic and Analytic Epidemiology, The University of Melbourne, Parkville, Victoria, Australia

## Introduction

We present recent findings based on the analysis of data from the Australasian Colorectal Cancer Family Study and the Colon Cancer Family Registry.

## *De novo* mutations

Carriers of a germline mutation in a DNA mismatch repair (MMR) gene, i.e. persons with Lynch syndrome, have substantially high risks of colorectal, endometrial, and several other cancers. The proportion of carriers who have *de novo* mutations (not inherited from either parent) is not known. Of 261 probands (202 clinic-based, 59 population-based) with MMR gene mutations for whom it was possible to determine the origin of the mutation, six (2.3%, 95%CI = 0.9–5.0%) were confirmed as *de novo* and the remaining 255 (97.7%, 95%CI = 95.0–99.1%) were inherited. Of the *de novo* mutation carriers, three were clinic-based probands (1.5%, 95%CI = 0.3–4.5%) and three were population-based probands (5.1%, 95%CI = 1.2–14.5%). Two were in *MLH1*, three in *MSH2*, and one in *MSH6*. These mutation carriers were recruited from family cancer clinics in Perth and Brisbane and via the Victorian Cancer Registry, Australia, and from Mayo clinic and via the Minnesota Cancer Surveillance System, USA. *De novo MMR* gene mutations are uncommon causes of Lynch syndrome.

## Cancer risks for non-carriers

To determine whether cancer risks for non-carriers of an MMR gene mutation from mutation carrying families are increased above that of the general population, we prospectively followed a cohort of 1,029 unaffected non-carriers. We estimated country-, age- and sex-specific standardized incidence ratios (SIRs) of different cancers for non-carriers. Over a median follow-up of 5 years, we found no evidence of non-carriers having an increased risk of any cancer, including colorectal cancer (SIR 1.02, 95%CI = 0.43 – 3.06, *P* = 0.97). These non-carriers had a colonoscopy screening for every four years.

## Second primary cancers

Apart from colorectal and endometrial cancers, risks of second primary cancers after a diagnosis of first primary colorectal cancer for MMR gene mutation carriers are yet to be established. Using a cohort of 764 carriers who had a diagnosis of colorectal cancer from the Colon Cancer Family Registry, we estimated age-, sex-, country- and calendar year-specific SIRs of second primary extracolonic cancers to compare with general population. We observed statistical evidence for significantly increased risks of cancers of the stomach (SIR=5.65), small intestine (SIR=72.75), liver (SIR=5.95), kidney (SIR=8.47), bladder (SIR=7.22), breast (SIR=1.85), brain (SIR=4.36), bone (SIR=17.99) and haemopoietic tissue (SIR=3.11) in both sexes, the prostate (SIR=2.05) in males, and the endometrium (SIR=40.34) and ovary (SIR=4.20) in females.

